# Multimodal treatment of pediatric patients with Askin’s tumors: our experience

**DOI:** 10.1186/s12957-018-1434-2

**Published:** 2018-07-13

**Authors:** Silvia Triarico, Giorgio Attinà, Palma Maurizi, Stefano Mastrangelo, Lorenzo Nanni, Vincenzo Briganti, Elisa Meacci, Stefano Margaritora, Mario Balducci, Antonio Ruggiero

**Affiliations:** 1 Paediatric Oncology Unit, A. Gemelli University Hospital, Catholic University of Sacred Hearth, Largo A. Gemelli, 8, 00168 Rome, Italy; 2 Pediatric Surgery Unit Gemelli University Hospital, Catholic University of Sacred Heart, Rome, Italy; 3 Pediatric Surgery Unit, San Camillo Forlanini Hospital, Rome, Italy; 4 Thoracic Surgery Unit, Gemelli University Hospital, Catholic University of Sacred Heart, Rome, Italy; 5 Radiotherapy Unit, Gemelli University Hospital, Catholic University of Sacred Heart, Rome, Italy

**Keywords:** Askin’s tumors, Thoracopulmonary region, Polychemotherapy, Radiotherapy, Surgery

## Abstract

**Background:**

We report our experience and outcomes about the management of Askin’s tumors [AT], which are rare primitive neuroectodermal tumors (PNETs) that develop within the soft tissue of the thoracopulmonary region, typically in children and adolescents.

**Methods:**

We retrospectively analyzed the charts of 9 patients affected by AT (aged 6–15 years), treated at the Paediatric Oncology Unit of Gemelli University Hospital in Rome between January 2001 and December 2016.

**Results:**

All nine patients underwent to biopsy followed by neoadjuvant chemotherapy. At the end of the neoadjuvant chemotherapy, they underwent to surgical removal of the residual tumor. Five patients with positive tumor margins and/or necrosis< 90% received local radiotherapy. Two patients with metastasis received an intensified treatment, with the addition of high dose adjuvant chemotherapy followed by peripheral blood stem cells rescue. No statistically significant correlation was found between outcome and gender; the presence of any metastasis and the radiotherapy. The overall survival was 65.14 months (95% confidence interval [95%CI], 45.81–84.48), and the 5 years survival was 60%, at a median follow-up of 53.1 months.

**Conclusion:**

Our study confirms that a multimodal treatment with surgery, chemotherapy, and radiotherapy may increase the survival in AT pediatric patients.

## Background

Primitive neuroectodermal tumors (PNETs) are malignant and aggressive “small round cells” neoplasms that arise from the primitive nerve cells of the central nervous system (central PNETs), but at times, they can affect any peripheral nerve (peripheral PNETs) in the neck, chest wall, retroperitoneum, pelvis, and extremities [1].

In 1979, Askin et al. described for the first time, 20 cases of children and adolescents affected by PNETs of the thoracopulmonary region [2]. Since then, PNETs developed within the soft tissue of the thoracopulmonary region are referred to as “Askin’s tumors” [AT]. In addition to this, AT occur typically in children and adolescents and they belong to the Ewing’s sarcoma (EW) family because of their histological, immunohistochemically, cytogenetic, and phenotypic similarities [3].

Because of the rarity of this neoplasm, the approach to AT is complex and it may require a multidisciplinary management [4].

In this study, we describe our experience with nine children and adolescents affected by AT, evaluating patients’ clinical characteristics, diagnosis assessment, multimodal treatment, and clinical outcomes.

## Methods

We performed a retrospective analysis of children and adolescents affected by AT who were treated at the Paediatric Oncology Unit of Gemelli University Hospital in Rome between January 2001 and December 2016.

Data collected included gender, age at diagnosis, clinical presentation, lactate dehydrogenase (LDH) levels, radiological findings, histopathology, management, and outcome.

At the admission, the patients showed different degrees of symptoms and clinical conditions. They underwent physical examination, biochemical, and radiological assessment. On all nine patients, a computed tomography (CT) scan was initially performed, which revealed the presence of the primitive chest mass. Subsequently, an 18F-flourodeoxyglucose positron emission tomography/computed tomography (PET-CT), a bone scintigraphy, abdomen ultrasonography (US) scan, and a bilateral osteomedullary biopsy were performed, in order to detect the presence of any distant metastasis.

All patients were treated with a multidisciplinary approach, with the option of chemotherapy, high-dose chemotherapy and autologous stem cell transplantation, surgery, and radiotherapy.

The diagnosis was achieved with an ultrasound or CT-guided biopsy. All patients received a polychemotherapy treatment based on the EuroEwing99 protocol [5], which consists of 6 cycles VIDE (vincristine 1.5 mg/kg/d at d1; ifosfamide 3 g/mq/d at d1, d2, and d3; etoposide 150 mg/mq/d at d1, d2, and d3; doxorubicine 20 mg/mq/d at d1, d2, and d3) and 8 cycles VAI (vincristine 1.5 mg/kg/d at d1; D-actinomycin 0.75 mg/mq/d at d1 and d2; ifosfamide 3 g/mq/d at d1 and d2).

At the end of the neoadjuvant chemotherapy, they underwent surgical removal of the residual tumor. The aim of the histopathological report was to assess necrosis and the absence of surgical positive margins. The goal of the surgery was the complete resection, defined as the absence of positive tumor margins and/or necrosis >  90% [6].

Local radiotherapy (44–54 Gy) was adopted for patients with positive tumor margins and/or tumoral necrosis <  90%. Furthermore, metastatic patients received an intensified treatment, with the addition of high-dose adjuvant chemotherapy with busulphan (3.75 mg/mq/d at d6, d5, d4, and d3) and melphalan (140 mg/mq at d2), followed by peripheral blood stem cells rescue (at d0).

All statistical analyses were performed using the statistical software *IBM SPSS© version 20.* Numerical variables are reported in mean and standard deviations (SD), whereas categorical variables are reported in numbers and percentages. Fisher’s exact test was used for testing the association between categorical dichotomic variables and the survival outcome in a univariate model. A value of *p < 0.05* two-sided was considered statistically significant. A Kaplan-Meier curve was performed to calculate overall survival at 5 years.

## Results

We reviewed data of nine patients affected by AT treated between January 2001 and December 2016. Patients’ characteristics, treatments, and evolutions are summarized in Table 1

**Table 1:** Patients’ characteristics, treatments, and follow-up

Patient gender	Age at diagnosis (years)	Tumor size (cm)	Tumor volume (cm^3^)	LDH (UI/L)	Metastasis	Neoadjuvant/adjuvant CT	RT	Relapse (months after remission)	Survival	Follow-up (months since diagnosis)
1 M	12	11	29.7	475	Yes	6 VIDE + 2 VAI, BUS/MELPH	No	No	Yes	125
2 M	6	6	62.4	460	No	6 VIDE + 8 VAI	Yes	No	No	11.4
3 F	13	7	211.1	461	No	6 VIDE + 8 VAI	Yes	No	Yes	170.9
4 M	14	15	1404	560	No	6 VIDE+ 8 VAI	Yes	No	Yes	76.5
5 M	9	9	345.8	510	No	6 VIDE + 8 VAI	No	Yes	No	35.6
6 F	15	4.5	166.4	210	No	6 VIDE + 8 VAI	Yes	No	Yes	104.2
7 F	10	16	457.6	320	No	6 VIDE + 8 VAI	Yes	No	No	29
8 M	11	7	117.9	280	No	6 VIDE + 8 VAI	No	No	Yes	28.7
9 F	6	5.5	50.5	420	Yes	6 VIDE + 2 VAI, BUS/MELPH	No	No	Yes	53.1

*M* male; *F* female; *VIDE* vincristine, ifosfamide, doxorubicine, D-actinomycin; *VAI* vincristine, D-actinomycin, ifosfamide; *BUS/MELPH* busulfan/melphalan; *RT* radiotherapy

There were 5 (55.6%) male patients and 4 (44.4%) females, with a male to female ratio of 1:1.25. Age at presentation ranged between 6 and 15 years old (median age, 11 years).

Patients were mostly symptomatic at the diagnosis. Seven of them (77.8%) presented a history of fever, with significant weight loss, cough, and dyspnea or chest pain; instead, the other two (22.2%) showed a swelling or palpable mass on the chest wall or in the supraclavicular region. These symptoms usually developed rapidly in 10–30 days.

**Fig. 1:**
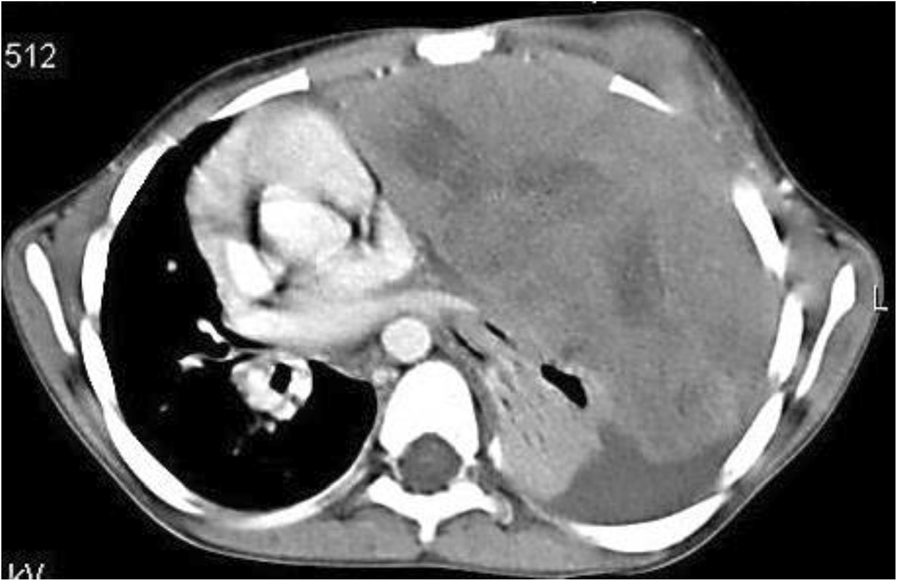
CT scan of a 10-year-old girl revealed a large lesion (mean diameter 16 cm) in the left hemithorax

Patients typically presented with a very large thoracic tumor, with a median diameter of 9 cm (range, 5.5–16). The main lesion was reported in the Fig. 1, which showed CT scan of a 10-year-old girl with a large lesion (mean diameter 16 cm) in the left hemithorax. The median volume was 166.4 cm^3^ (range 29.7–1404). In all nine patients, one or more ribs were involved, typically with erosion. Four patients (44.4%) showed the involvement of soft tissues. Two (22.2%) of these patients were metastatic at the bone, bone scintigraphy, and PET-CT detected bone metastasis. Bone marrow infiltration was absent in all these patients.

Laboratory investigation were normal in all cases, except for an increased LDH in 8 patients (88.9%, median value 460 UI/l; range 210–560).

After a tumor biopsy, which typically revealed the presence of “small round cell tumour” positive for neuron-specific enolase (NSE) and for CD99, all patients received neoadjuvant chemotherapy, and subsequently, they underwent surgery for tumor resection. Five patients (55.6%) with positive tumor margins and/or necrosis < 90% received local radiotherapy (44–54 Gy). Two patients (22.2%) with metastatic disease received an intensified treatment, with the addition of high-dose adjuvant chemotherapy, including busulphan and melphalan, followed by peripheral blood stem cells rescue.

Seven patients (77.8%) were disease-free at the end of the treatment, including the two metastatic patients. Two non-metastatic patients (22.2%) showed a disease progression during the adjuvant chemotherapy, and finally, they died.

They died from pulmonary edema and insufficiency of respiration due to pulmonary metastatic disease.

Recurrent disease was reported in one non-metastatic patient (11.1%) 7 months after the end of the treatment. This patient underwent second-line chemotherapy, without going into remission, and he finally died due to disease progression and finally for septic shock.

**Fig. 2:**
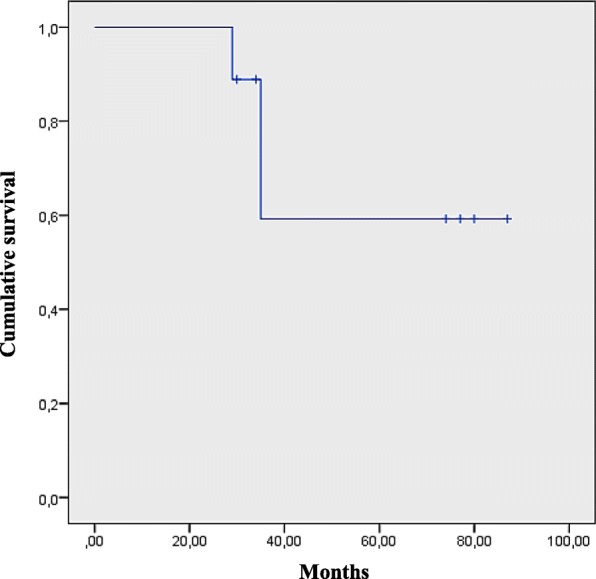
5 years overall survival of our patients

We found an overall survival of 65.14 months (95% confidence interval [95% CI], 45.81–84.48) and a 5-year survival of 60%, at a median follow-up of 53.1 months, as exposed in the Fig. 2.

It was not found that there is a statistically significant correlation between the survival and the gender, the presence of any metastasis, and the radiotherapy, as Table 2 shows.

**Table 2:** Correlation between the outcome and the sex, the presence of any metastasis, and the radiotherapy

*–*	No. of patients	% value	Survival outcome	*p* value
Sex				*1.000*
Male	5	55.6%	3	
Female	4	44.4%	3	
Metastasis				*0.500*
Yes	2	22.2%	2	
No	7	77.8%	4	
RT				*0.524*
Yes	5	55.6%	4	
No	4	44.4%	2	

The significance level was determined at *p*<0.05

## Discussion

AT belong to the family of Ewing’s sarcoma (ES), which includes tumors of neuroectodermal origin that occur in bone and soft tissues (classical osseous ES, atypical extraosseous ES, PNET, and AT) [7]. AT are histologically identical to other PNETs, but they typically originate in the chest wall. They are the most common malignant chest wall tumors in children and young adults, followed by rhabdomiosarcoma, lymphoma, fibrosarcoma, osteosarcoma, and chondroma, as reported by Shamberger et al. [6, 8].

These neoplasms can develop at any age, but they are extremely rare and they occur more during the pediatric age than in adults [9]. Among our 9 patients, the younger was 6 years old and the oldest was 15 years old, and no significant difference was found in the prevalence between males or females.

Commonly, patients present a palpable and usually painful mass in the chest wall and/or fever, weight loss, cough, dyspnea, and even severe respiratory distress. From biochemical investigations, values of LDH are usually greater than normal and that seems to be associated with a worse prognosis [10].

Patients usually undergo radiological examination, such as X-rays and/or chest CT scans. The masses detected by chest CT scans are typically heterogeneous, with necrosis or cystic degeneration and hemorrhage, but rarely with calcifications, with a tendency to displace the adjacent organs and produce pleural effusions or rib erosions. CT scans are very useful for evaluating tumor extension during diagnosis, as well as for establishing the effects of chemotherapy and assessing tumor recurrence. Nevertheless, Sallustio et al. show that magnetic resonance imaging (MRI) may be better than CT for evaluating the tumor’s local invasion and extension to pleura, lung, or diaphragm. Consequently, MRI may be considered complementary to CT scans especially for the evaluation of the extension of very large chest wall tumors [11].

Distant metastases are rare in AT, but they may occur in the lymphonodes, extrathoracic skeleton, liver, brain, retroperitoneum, bone marrow, and adrenal glands. PET-CT may be helpful for the characterization of the metabolic activity of the tumor and for the detection of distant metastases [12, 13].

The typical histological feature is the presence of small round cells, with Homer-Wright rosettes and immunohistochemical positivity for several neural markers, such as NSE and CD99. The identification of chromosomal translocation t(11;22)(q24,q12) and the detection of proto-oncogenes (n-myc, c-myb, c-ets-1) may be additional diagnostic criteria [14].

The prognosis of AT reported in the literature is very poor. In our experience, we did not find a significant correlation between the outcome and the categorical variables analyzed (gender, the presence of any metastasis, and radiotherapy). In addition to this, we observed that a prompt diagnosis and an intensive treatment are crucial for the patients’ survival.

A complete surgical resection with wide margins can give better prospects of survival; however, this is not always possible due to the anatomical complexities of the chest. As a consequence, surgery must always be associated with chemotherapy (including ifosfamide or cyclophosphamide, vincristine, etoposide, D-actinomycin, and doxorubicine) and radiotherapy (usually 44–54 Gy) [15].

Neoadjuvant chemotherapy may have important benefits, such as the chance of treating distant metastases, a lower risk of intraoperative tumor rupture and tumor cells dissemination, and an enhanced probability of less extensive surgery as well as preserving a better post-operative function [16, 17]. In their study, Bacci et al. demonstrated that, in cases of surgically treated patients with non-metastatic EW, the most important prognostic factor seems to be neoadjuvant chemotherapy-induced necrosis, with better clinical outcomes in patients with good necrosis (90%) [18, 19].

Several studies showed that the poor prognosis of high-risk EW patients (including metastatic patients) may be improved by high-dose chemotherapy with busulfan and melphalan followed by peripheral blood stem cell transplantation [20].

Radiotherapy is an active modality for assuring local control, used as definitive radiotherapy in inoperable tumors or in combination with surgery (either pre- or post-surgery). Definitive radiotherapy is performed in inoperable lesions only. Inoperability is given in large tumors that cannot be completely resected and in tumors in critical sites where complete surgery would be mutilating or associated with a high risk of severe complications. The definitive radiotherapy is to start following course 6 of the induction regimen for patients in the conventional arms, or 8–10 weeks after stem cell reinfusion in patients of the high-dose chemotherapy with busulfan and melphalan [5, 21].

The results of the study conducted on eight patients with AT by Christianses et al. evidenced that AT require an aggressive multimodal treatment, consisting of pre- and post-operative chemotherapy, radical surgical resection, and postoperative irradiation [22]. In addition to this, in a study conducted among 104 patients with AT, Laskar et al. demonstrated that the combination of neoadjuvant and adjuvant chemotherapy, surgery, and radiotherapy resulted in optimal outcome [23].

## Conclusions

In our study, we confirm that surgical resection is essential for the treatment of AT pediatric patients. Moreover, a multimodal management, including surgery, chemotherapy, and radiotherapy, should be used for improving the overall survival.

## Abbreviations

AT: Askin’s tumors
CI: Confidence interval
CT: Computed tomography
ES: Ewing’s sarcoma
LDH: Lactate dehydrogenase
MRI: Magnetic resonance imaging
NSE: Neuron-specific enolase
PET-CT: Positron emission tomography/computed tomography
PNETs: Primitive neuroectodermal tumors
US: Ultrasonography
VAI: Vincristine, D-actinomycin, ifosfamide
VIDE: Vincristine, ifosfamide, doxorubicine, etoposide
