# Correction to: Multimodal treatment of pediatric patients with Askin’s tumors: our experience

**DOI:** 10.1186/s12957-018-1464-9

**Published:** 2018-09-25

**Authors:** Silvia Triarico, Giorgio Attinà, Palma Maurizi, Stefano Mastrangelo, Lorenzo Nanni, Vito Briganti, Elisa Meacci, Stefano Margaritora, Mario Balducci, Antonio Ruggiero

**Affiliations:** 1 Paediatric Oncology Unit, A. Gemelli University Hospital, Catholic University of Sacred Hearth, Largo A. Gemelli, 8, 00168 Rome, Italy; 2 Pediatric Surgery, Unit Gemelli University Hospital, Catholic University of Sacred Heart, Rome, Italy; 3 Pediatric Surgery Unit, San Camillo Forlanini Hospital, Rome, Italy; 4 Thoracic Surgery Unit, Gemelli University Hospital, Catholic University of Sacred Heart, Rome, Italy; 5 Radiotherapy Unit, Gemelli University Hospital, Catholic University of Sacred Heart, Rome, Italy

## Correction

In the original article mentioned above, the name of the sixth author was wrongly mentioned as “Vincenzo Briganti” instead of “Vito Briganti”.

The original article has been corrected.
